# Knowledge and Practice of Gynecologists About Tdap and Influenza Vaccination: A Cross-Sectional Study

**DOI:** 10.7759/cureus.40037

**Published:** 2023-06-06

**Authors:** Harpreet Kaur, Alka Sehgal, Nisha Malik, Sushruti Kaushal, Asmita Kaundal

**Affiliations:** 1 Obstetrics and Gynecology, All India Institute of Medical Sciences, Bilaspur, Bilaspur, IND; 2 Gynaecology, Government Medical College and Hospital, Chandigarh, IND

**Keywords:** immunisation in pregnancy, td vaccination, influenza, vaccination in pregnancy, tdap

## Abstract

Background: Infants are vulnerable to diphtheria and pertussis in their early months. In this initial period, maternally derived antibodies provide significant protection to newborns. Similarly, influenza poses a significant risk of morbidity and mortality for pregnant mothers and infants. It has been observed that, despite the evident recommendations, the uptake of these vaccines is still not optimal.

Methodology: The current study was undertaken as a cross-sectional survey among the practicing gynecologists of North India voluntarily. A structured questionnaire was made available online to 300 practicing gynecologists either on their WhatsApp or email addresses. The data were compared based on urban and rural practices. A record was also made of the participants’ type of practice setup, e.g., working in a primary health setting, a district hospital, or a teaching institute.

Results: Of the 148 participants who responded to the survey, 45.3% and 64.2%, respectively, administered influenza and Tdap vaccines to their patients. The main barriers cited by the respondent doctors were the non-affordability, non-availability, and non-inclusion of vaccines in the national immunization program and a lack of awareness among the practitioners (Spearman correlation 0.4; p<0.000).

Conclusion: The results of this survey suggest that increasing awareness among gynecologists and the public and improving the availability of vaccines and their inclusion in the national program could most likely increase the practice of the recommendation or administration of the Tdap vaccine in pregnant females.

## Introduction

Infants less than six months of age are vulnerable to pertussis. There have been reappearing pockets of diphtheria in developing countries in recent years. In India, many incidences of pertussis among neonates have been reported recently, indicating that infant pertussis is not so uncommon in the country [1,2]. Many times, deaths related to pertussis in infants occur at less than two months of age. The infants acquire immunity from their vaccination series after that period; however, during this initial vulnerable period, it has been observed that maternally derived antibodies offer significant protection to the newborn from pertussis [3]. Maternal vaccination with tetanus toxoid, reduced diphtheria toxoid, and acellular pertussis (Tdap) can protect infants in the initial days of life. Infants born to mothers who have received the Tdap vaccination during pregnancy have a lower risk of pertussis and related complications [3].

Similarly, infants less than six months of age are at high risk of influenza-related illness. Moreover, influenza during pregnancy can lead to serious maternal morbidity and mortality. Influenza vaccination for pregnant mothers can reduce the risk of influenza-related illness and hospitalization in newborn infants and their mothers [4].

The advisory committee on immunization practices (ACIP) in the USA recommends that all pregnant women receive influenza vaccination, irrespective of the trimester of pregnancy [5]. Tdap vaccination is recommended in all pregnancies, preferably during the 27-36 weeks period of gestation. Various international guidelines, such as those from Public Health England, the American College of Obstetricians and Gynecologists (ACOG), and the Society of Obstetricians and Gynecologists of Canada (SOGC), recommend tetanus toxoid, reduced diphtheria toxoid, and acellular pertussis (Tdap) vaccination during each pregnancy to provide maximal protection to every infant [3,6]. In India, the Tdap vaccination during pregnancy is recommended by both Federation of Obstetrics and Gynecological Societies of India (FOGSI) and the Indian Academy of Pediatrics (IAP) [7].

Despite these guidelines and recommendations, there have been wide variations in the delivery of the Tdap vaccination during pregnancy. The uptake of both Tdap and influenza vaccinations in pregnancy has not been very encouraging. Approximately 32% of women received vaccinations for both influenza and Tdap, 15% for influenza, and 21.6% for Tdap, as per a recent CDC survey [8]. There are different reasons for the suboptimal uptake, for instance, inadequate knowledge of practitioners regarding guidelines and recommendations, issues related to the availability of vaccines, accessibility and affordability of vaccines, and a lack of awareness among healthcare workers and pregnant women [9,10]. 

Knowledge, attitude, and practice (KAP) of obstetricians could be key factors that can influence the uptake of vaccinations in pregnancy [3]. Assessing the attitude and acceptability of gynecologists, with their role as the main implementor, is important to identify perceived barriers and opportunities for optimal implementation.

Keeping these observations in view, the current study aims to study the knowledge and attitude of obstetricians toward Tdap and Influenza vaccination. An attempt to create awareness amongst them about the importance of these vaccines in pregnancy was also made via this study.

## Materials and methods

Objectives

To assess the knowledge, attitude, and practice (KAP) of gynecologists about Tdap and influenza vaccination during pregnancy and to create awareness among practicing gynecologists about Tdap and Influenza vaccination.

Methodology

The current study has been conducted through a survey based on a structured questionnaire made available online to the practicing gynecologists of north India, either on their WhatsApp or email addresses, to seek their response on the issue under investigation. Participation was voluntary. The questionnaire comprised three parts. The consent of the respondents to participate in the survey was obtained through the first part of the questionnaire. The subsequent part aimed at assessing their knowledge, practice, and attitude towards Tdap and influenza vaccination in pregnancy. The last part of the questionnaire was focused on judging their knowledge about the significance of these vaccines in pregnancy.

The data was distributed in the form of Google Forms (California, US) and collected anonymously by designated staff. The information collected for this purpose included the number of years of practice, the location of practice, primary qualification, type of practice setup, and barriers to the implementation of this vaccine in pregnancy. The suggested measures to overcome these barriers were recorded for those already implementing the Tdap vaccination; an attempt was made to know how they created awareness and implemented the method.

Ordinal data, like responses, were recorded on a 3-point Likert scale. The data were compared based on urban and rural practices. A record was also made of the participants' type of practice setup, e.g., working in a primary health setting, a district hospital, or a teaching institute. The validity of the content questionnaire was checked by three subject experts before distribution. The study was approved by the institute's ethics committee.

Duration of the study

The data was collected online from the respondent practicing gynecologists during the period of 15^th^ September to 15^th^ October 2021.

Intervention

No intervention was made.

Statistical methods

The numerical data are presented as percentages, and the results are presented through graphs, bar diagrams, or numerical values. The continuous variables are presented as means ± SD, and the categorical variables as percentages. Further, stratified analysis has been done to assess potential associations between performing vaccination and demographics and perceived barriers. P-values < 0.05 were considered statistically significant. Percentages were calculated using Microsoft Excel version 16.51 (Redmond, USA). 

Sample size calculation and sampling procedure

Our target population consisted of the gynecologists of North India registered with the FOGSI Society. The questionnaire was shared with them through WhatsApp and email.

At a confidence level of 90%, the minimum number of participants needed from the target population of gynecologists in North India to determine an expected frequency of 50% at an absolute precision of 5% was calculated to be 277 using OpenEpi [11]. The questionnaire was distributed to 300 practicing gynecologists in North India (https://www.openepi.com/SampleSize/SSPropor.htm).

## Results

**Figure 1:**
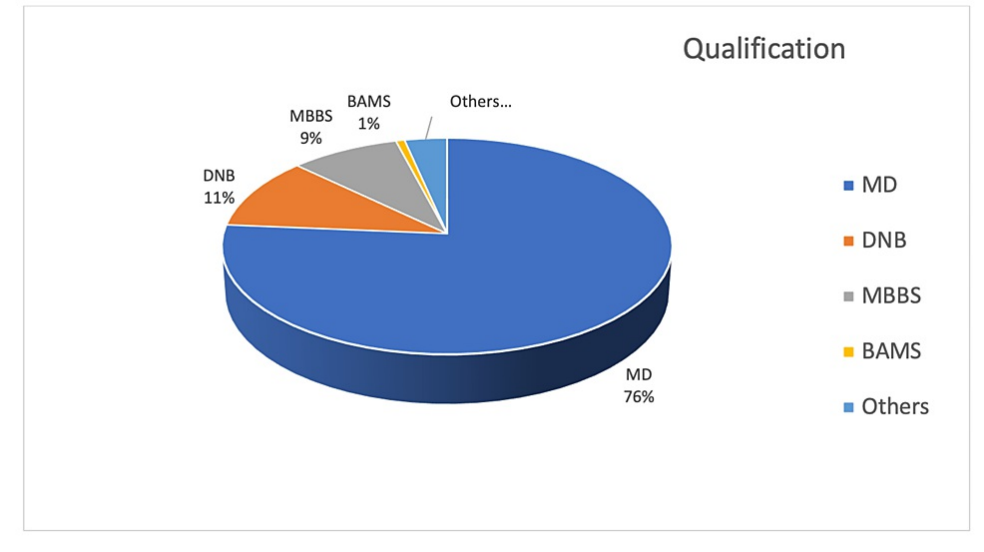
Education-wise distribution of the respondents (148 responses)

The questionnaire was distributed to 300 practicing gynecologists in North India. A total of 148 gynecologists responded to the survey questionnaire. Figure 1 shows the education-wise distribution of the respondents. The majority of them (87%) were specialists in obstetrics and gynecology (MD or DNB in Obstetrics and Gynecology). The rest were medical graduates (MBBS/BAMS) practicing obstetrics and gynecology. 

**Figure 2:**
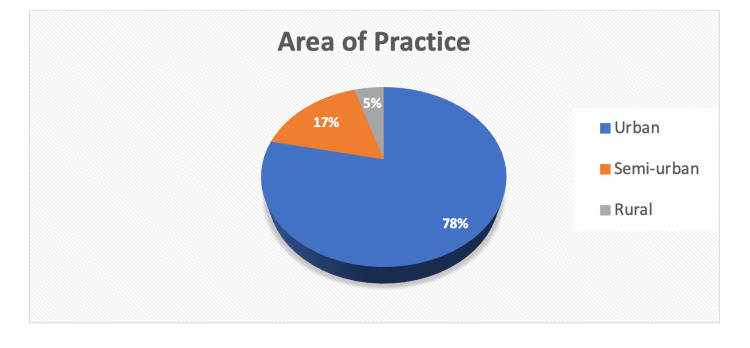
Distribution of respondents by area of practice

Figure 2 represents the area-wise distribution of the respondents. 

Almost 31% of the participants had more than 20 years of experience in gynecology practice, and only eight participants, i.e., 5%, had less than five years of experience in gynecology services.

**Figure 3:**
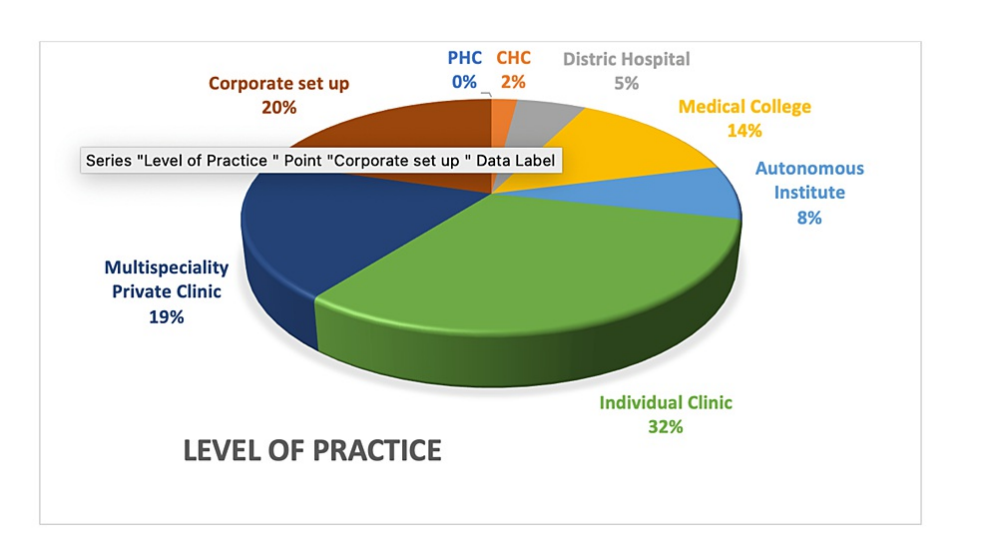
Distribution of respondents by their level of practice

A record was made of their level of practice and their practice for Tdap vaccination. Figure 3 describes the distribution of practitioners by their level of practice.

**Figure 4:**
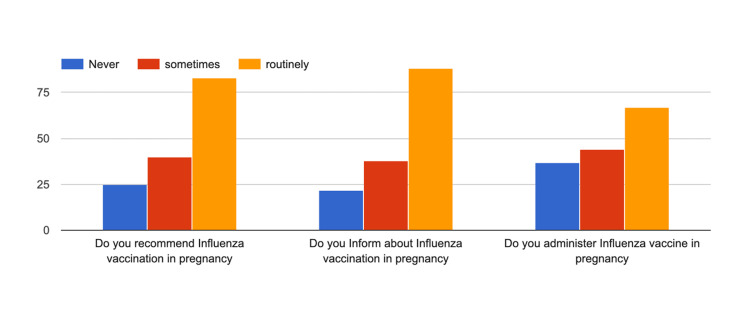
Practice of respondents for influenza vaccination

**Figure 5:**
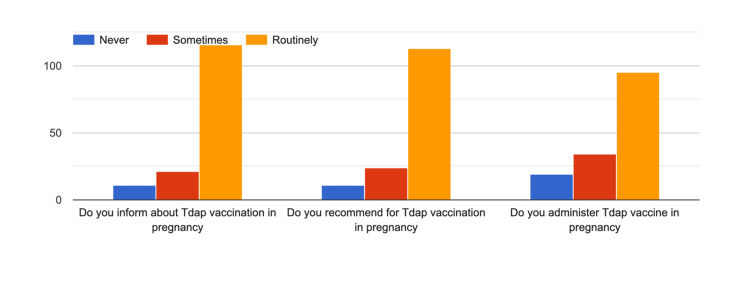
Practice of respondents for Tdap vaccination

Regarding the practice of influenza vaccination, though 60% of respondents routinely informed their patients, only 45.3% routinely administered the influenza vaccine to their patients. Further, there existed a gap between the information provided for Tdap routinely and the actual practice of administering the Tdap vaccine, with respective percentages of 78.4 and 64.2 (Figures 4, 5).

The majority of the practitioners (83.8%) themselves created awareness among the antenatal women for the vaccination, while in the remaining cases, this job was performed by either a nurse or a counselor. More than three-fourths of the participants (81%) became aware of the Tdap vaccination during pregnancy during the last five years only, while 15% of practitioners had information about this vaccination during the last 5-10 years. The major source of their learning was either CMEs and conferences or workplace academics.

While assessing the knowledge of practitioners about the practice of Tdap in pregnancy, 81% of practitioners answered correctly that the ideal time of administering the vaccine was between the 27th and 36th weeks of pregnancy, while 13.5% of participants felt that it could be given after the first trimester of pregnancy, and 2.7% considered it anytime in pregnancy. As many as 94% of participants had the correct perception that the injection needed to be given in each pregnancy. Only 19% of practitioners knew about the approximate cost of the vaccine, and the rest were either unaware or had a wrong perception of the cost.

**Figure 6:**
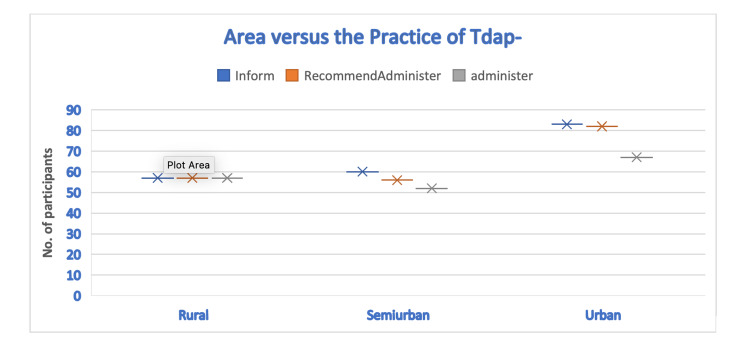
Area-wise distribution versus practice of Tdap among the practitioners

When the practice of administering the Tdap vaccine was analyzed separately for each area of practice, the practitioners in urban areas prescribed the vaccine most frequently to their patients. However, those from semi-urban areas prescribed the vaccine more frequently than rural practitioners. Overall, the results were found to be statistically non-significant (Figure 6).

**Figure 7:**
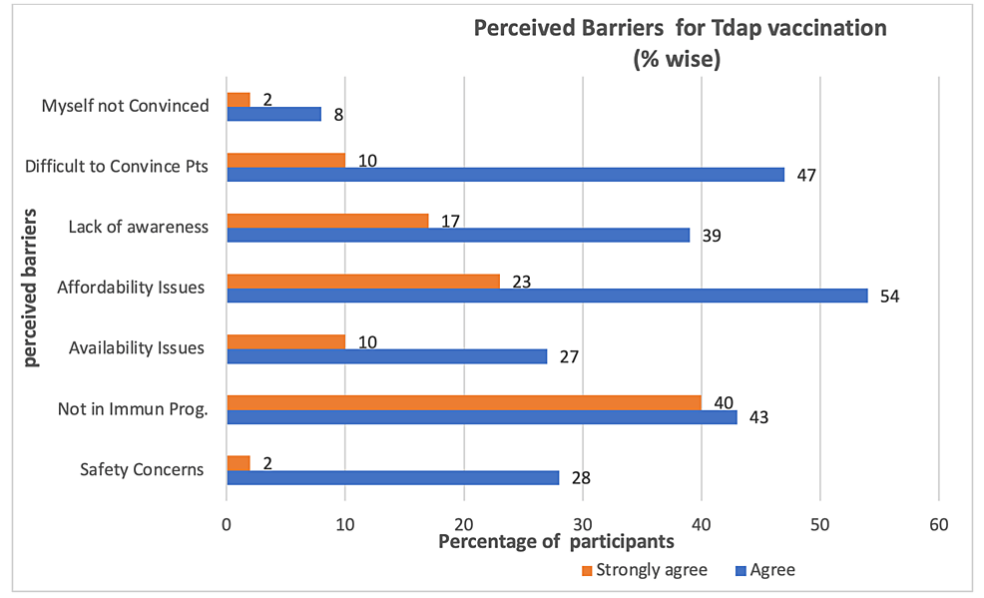
Perceived barriers to Tdap practice

The most common barriers in the administration of this vaccine, as described by the respondent doctors, are non-affordability by the patients, non-inclusion of the vaccine in the national immunization program, non-availability of the vaccine, lack of awareness among the practitioners, and difficulty convincing the antenatal women for the vaccine (Figure 7).

**Figure 8:**
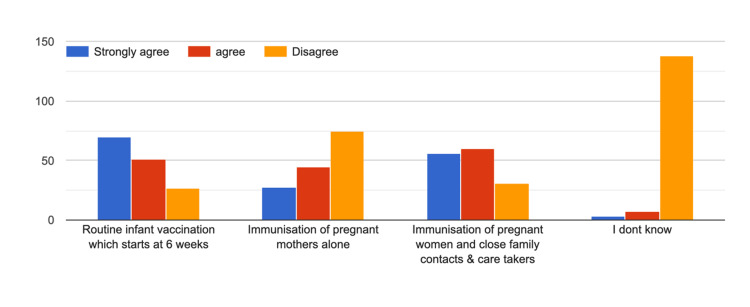
Practitioners' knowledge regarding what protects newborns from pertussis

**Figure 9:**
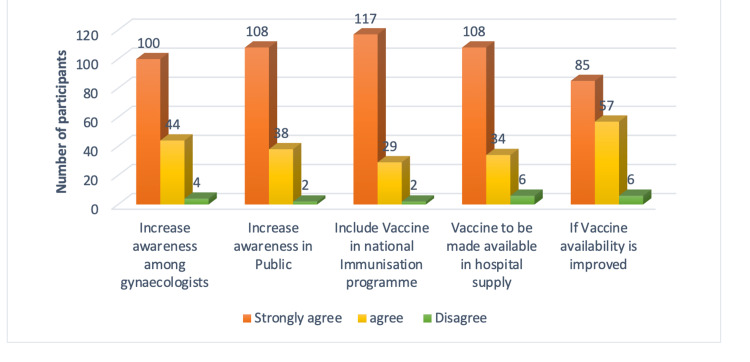
Participants’ suggestions to improve Tdap vaccination in pregnancy

Further, Figure 8 represents the practitioner's knowledge, and Figure 9 represents their suggestions to improve the vaccination of pregnant women.

Correlation analysis

It was observed that the gynecologists who recommend the influenza vaccine were more likely to recommend the Tdap vaccine as well (Spearman correlation 0.6; p< 0.000). Among the perceived barriers, the availability of the Tdap vaccine was found to be correlated with the clinician’s inclination to inform/recommend/administer the Tdap vaccine (Spearman correlation 0.4; p<0.000). Availability of the Tdap vaccine and difficulty in convincing patients were identified as the perceived barriers against recommendation or administration of the Tdap vaccine (Spearman correlation 0.43; p<0.000; Spearman correlation 0.44; p<0.000), and it was found that lack of conviction among the gynecologists was also a barrier to the lack of recommendation of the Tdap vaccine (Spearman correlation 0.42; p<0.000).

## Discussion

It has been observed that the uptake rate of Tdap and influenza vaccines is much lower despite clear recommendations. There are many barriers to the uptake of these vaccines, including a lack of awareness on the part of women and families and of obstetricians’ practices and knowledge regarding these vaccines [8-10]. A survey recently conducted by the Centre for Disease Control and Prevention (CDC) on pregnant women reported that only 32.8% of such women were vaccinated with both vaccines. As many as 15.3% of these women reported that they were not offered these vaccines during pregnancy. The knowledge and practice of obstetricians can play an important role in the vaccination of pregnant women [11].

The current study was undertaken to understand the knowledge and practice of gynecologists regarding Tdap and the influenza vaccine. The majority of respondents in the study belonged to urban settings and had post-graduate experience in obstetrics and gynecology. Relatively more practitioners from the urban area were administering the Tdap vaccine to their patients, though the difference was not statistically significant. The majority of the respondents were from urban areas, as participation in the survey was on a voluntary basis. The questionnaire was made available to the respondent practitioners via their WhatsApp or email addresses. 

The study pointed out that a gap existed between the actual practice of vaccine administration and the information provided to antenatal women regarding the vaccine. The main reason cited by the practitioners for the low vaccine coverage was the non-availability of the vaccine. Non-affordability was another reason for it. Another important barrier in the way of vaccine administration was the lack of awareness among gynecologists regarding its benefits in pregnancy, the correct timing of administration, and the cost. Many practitioners failed to emphasize the importance of this vaccine to their patients. They themselves lack conviction regarding the importance of Tdap vaccination during pregnancy. In a study conducted by Thain et al., the main barriers cited by local obstetricians were uncertainty about recommendations and a lack of time for patient education. Thus, there is a need to raise awareness of evidence-based recommendations among gynecologists, which can be achieved through seminars, or webinars, or conferences [12]. In an Italian study, the vaccination rates for these two vaccines during pregnancy were dismal. Among the women surveyed, just 5.6% reported being vaccinated against influenza, and 16.4% received Tdap in their current or previous pregnancy. From a small sample of gynecologists surveyed in this study, the main reasons cited for the low level of vaccination were concerns about vaccine safety, a lack of awareness, and a general misconception about the necessity of vaccination. To overcome these barriers and increase vaccine uptake, it is of paramount importance to increase the awareness level of gynecologists about this vaccine [13].

In a study conducted among German Gynecologists, those who themselves received the influenza vaccine were able to assure pregnant women regarding both the influenza and pertussis vaccines. Thus, the physician's own confidence and practice regarding the vaccine can play a significant role in encouraging their patients [14].

The current study, which focused on further stratification to find an association between the area of practice and the perceived barriers, failed to find any significant association. Similarly, the gynecologists’ practice of vaccination was not significantly affected by their duration of practice.

On the basis of the findings of this study, it can be suggested that the inclusion of these vaccines in the national immunization program can help increase vaccine coverage and create awareness among physicians and the public at large. Increasing awareness has been cited as the main strategy to improve vaccination coverage in pregnancy in earlier studies as well [15].

The ACOG practice bulletin has highlighted the need for Tdap vaccination in pregnancy for the protection of newborn babies in the early vulnerable period, and for that reason, raising awareness among gynecologists is of utmost importance to improve vaccine coverage [1].

The results of this survey suggest that increasing awareness among gynecologists and the public and improving the availability of vaccines and their inclusion in the national program could most likely increase the practice of recommending or administering the Tdap vaccine to pregnant females.

Limitations of the study

As the survey was conducted online through a questionnaire on voluntary participation, there was unequal participation between urban and rural practitioners. Further, the response data used for this study may have been biased due to respondents’ differences of opinion on the issue under investigation. The framework used for the survey may not be representative of the whole population due to variations in response rates. A smaller sample size than calculated due to non-response might affect the validity of the results. 

Strength of the study

To the best of our knowledge, this is the first study to address the issue of Tdap vaccination among pregnant women in India. We have reported the correlation between different variables affecting the uptake of Tdap vaccination.

## Conclusions

Tdap and influenza vaccinations in pregnancy are important for the protection of newborn babies in the early neonatal period to reduce serious morbidity and mortality by inducing maternally acquired antibodies. Despite clear recommendations, vaccination uptake is not optimal in pregnancy, and gynecologists’ practices and knowledge can play a major role in this regard. There is a need to improve the awareness of gynecologists regarding these vaccines. Vaccines should be made easily available, which can go a long way toward protecting newborn babies from these life-threatening illnesses in early neonatal life.
